# A feature extraction framework for discovering pan‐cancer driver genes based on multi‐omics data

**DOI:** 10.1002/qub2.40

**Published:** 2024-04-05

**Authors:** Xiaomeng Xue, Feng Li, Junliang Shang, Lingyun Dai, Daohui Ge, Qianqian Ren

**Affiliations:** ^1^ School of Computer Science Qufu Normal University Rizhao China

**Keywords:** cancer driver genes, feature extraction, multi‐omics data, network propagation, pan‐cancer

## Abstract

The identification of tumor driver genes facilitates accurate cancer diagnosis and treatment, playing a key role in precision oncology, along with gene signaling, regulation, and their interaction with protein complexes. To tackle the challenge of distinguishing driver genes from a large number of genomic data, we construct a feature extraction framework for discovering pan‐cancer driver genes based on multi‐omics data (mutations, gene expression, copy number variants, and DNA methylation) combined with protein–protein interaction (PPI) networks. Using a network propagation algorithm, we mine functional information among nodes in the PPI network, focusing on genes with weak node information to represent specific cancer information. From these functional features, we extract distribution features of pan‐cancer data, pan‐cancer TOPSIS features of functional features using the ideal solution method, and SetExpan features of pan‐cancer data from the gene functional features, a method to rank pan‐cancer data based on the average inverse rank. These features represent the common message of pan‐cancer. Finally, we use the lightGBM classification algorithm for gene prediction. Experimental results show that our method outperforms existing methods in terms of the area under the check precision‐recall curve (AUPRC) and demonstrates better performance across different PPI networks. This indicates our framework’s effectiveness in predicting potential cancer genes, offering valuable insights for the diagnosis and treatment of tumors.

## 1 INTRODUCTION

Cancer ranks as a leading cause of death and poses a significant public health challenge [1, 2]. It is recognized as a heterogeneous disease, driven by a variety of genomic, epigenomic, and transcriptional genomic alterations, including single nucleotide variants, gene expression changes, DNA methylation and chromosomal abnormalities [3, 4]. Recent advancements in next‐generation sequencing technologies [5], exemplified by the efforts of the International Cancer Genome Consortium and The Cancer Genome Atlas (TCGA) [6, 7], have produced an extensive collection of multi‐omics data (somatic mutations, DNA methylation, copy number variation, etc.). The study of cancer driver genes, facilitated by resources such as the Network of Cancer Genes (NCG) and the COSMIC Cancer Gene Census (CGC), has made significant contributions to our understanding [8, 9]. Therefore, the effective identification of cancer driver genes, leveraging the aforementioned data, is crucial for advancing cancer research and treatment strategies [10].

Recent methods for identifying cancer driver genes have primarily focused on mutation frequencies [11]. For example, MutSigCV identifies driver genes by calculating mutation frequencies across all samples, targeting those with recurrent mutations [12]. However, this method overlooks oncogenes with low mutation frequencies. The 20/20+ method [13] employs random forest (RF) algorithms and somatic mutation data to predict oncogenes and tumor suppressor genes but considers only the somatic mutation information, neglecting other valid information. In addition, gene signaling, regulation, and protein complex interactions, encapsulated within protein–protein interaction (PPI) networks, are equally crucial in the identification of cancer genes [10, 14]. For example, PageRank [15] utilizes PPI networks to rank genes based on their likelihood of cancer driver genes but ignores the biological features of genes. HotNet2 [16], a directed heat diffusion model, identifies mutated cancer gene modules by clustering mutations of known cancer pathways, yet it relies solely on mutation data as biological features. Deep learning methods such as DeepWalk [17] learn network node features. However, few methods are available to combine the multidimensional genetic features of multi‐omics data with the structural features of protein interaction networks [18]. Examples of such include EMOGI [19], a graph convolutional network (GCN)‐based model [19], that combines multidimensional multi‐omics gene features, including genomic [19], epigenomic, and transcriptional data with PPI networks to identify cancer genes, offering improved driver gene identification. However, the multidimensional gene signature of EMOGI is only simply preprocessed, utilizing some biological data as gene features without considering the topological features of genes, and failing to extract more effective information from gene features. To address these limitations, we propose a feature extraction framework for discovering pan‐cancer driver genes based on multi‐omics data (MVKTS).

MVKTS uses multidimensional multi‐omics gene features as node features of PPI networks, employing a network propagation algorithm to correlate gene functions, particularly for genes with weak node information [14]. This process yields functional features of genes, from which we further extract enhancement features to assist in the prediction of pan‐cancer driver genes. Initially, we derive distribution features from the functional features—mean, variance, and kurtosis of pan‐cancer genes—to analyze the concentration trend, dispersion degree, and distribution pattern of multidimensional multi‐omics data. Subsequently, we extract TOPSIS (technique for order preference by similarity to ideal solution) features from the functional features [20]. TOPSIS is a method to extract features using the ideal solution [21, 22], ranking pan‐cancer genes based on the information from 16 cancer types and effectively highlighting differences among various cancer types. Additionally, SetExpan features, derived from the functional features as enhanced features [23], utilize an unsupervised ranking approach to sort data from 16 cancer types for each omics by averaging the inverse ranking, thus scoring each gene [23]. Finally, the lightGBM algorithm is used for the classification prediction of pan‐cancer driver genes [24, 25]. Comparative results across six PPI networks show that MVKTS achieves higher precision‐recall (PR) curve (AUPRC) values than other identification methods, indicating its efficiency in predicting pan‐cancer cancer driver genes.

## 2 RESULTS

### 2.1 Overview of MVKTS

**FIGURE 1:**
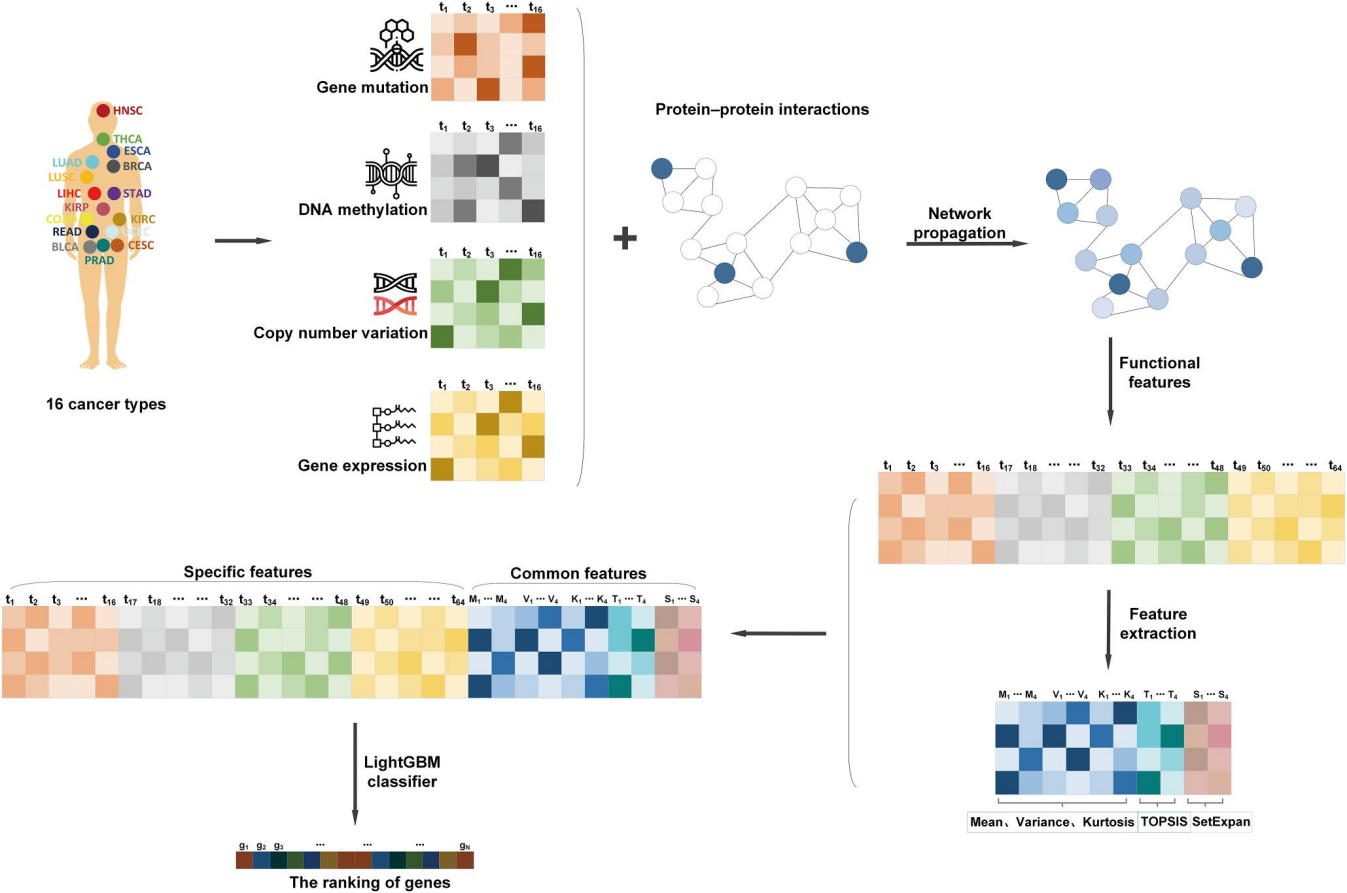
Framework of the MVKTS. (i) Mutation, DNA methylation, copy number variation, and gene expression data are collected for 16 cancer types. (ii) Functional features of genes are obtained by combining multi‐omics data with PPI networks using a network propagation algorithm. (iii) Distribution features, TOPSIS features, and SetExpan features corresponding to the omics data are extracted from the functional features of the genes. (iv) Final gene ranking is obtained by joining the four features and using the lightGBM classifier.

We propose a framework for pan‐cancer driver gene feature extraction based on multi‐omics data (Figure 1). We first collect mutation, DNA methylation, copy number variation, and gene expression data from TCGA for 29,446 samples corresponding to 16 cancer types [7]. Each type of omics data undergoes preprocessing before being integrated with one of the six PPI networks through a network propagation algorithm to obtain the functional features of the genes [14]. Subsequently, we extract distribution features [26], TOPSIS features, and SetExpan features of the corresponding omics data from the functional features of the genes [20, 21, 23]. Finally, we obtain the ranking of genes using the lightGBM classifier [24].

### 2.2 Performance comparison of MVKTS for predicting pan‐cancer driver genes

**FIGURE 2:**
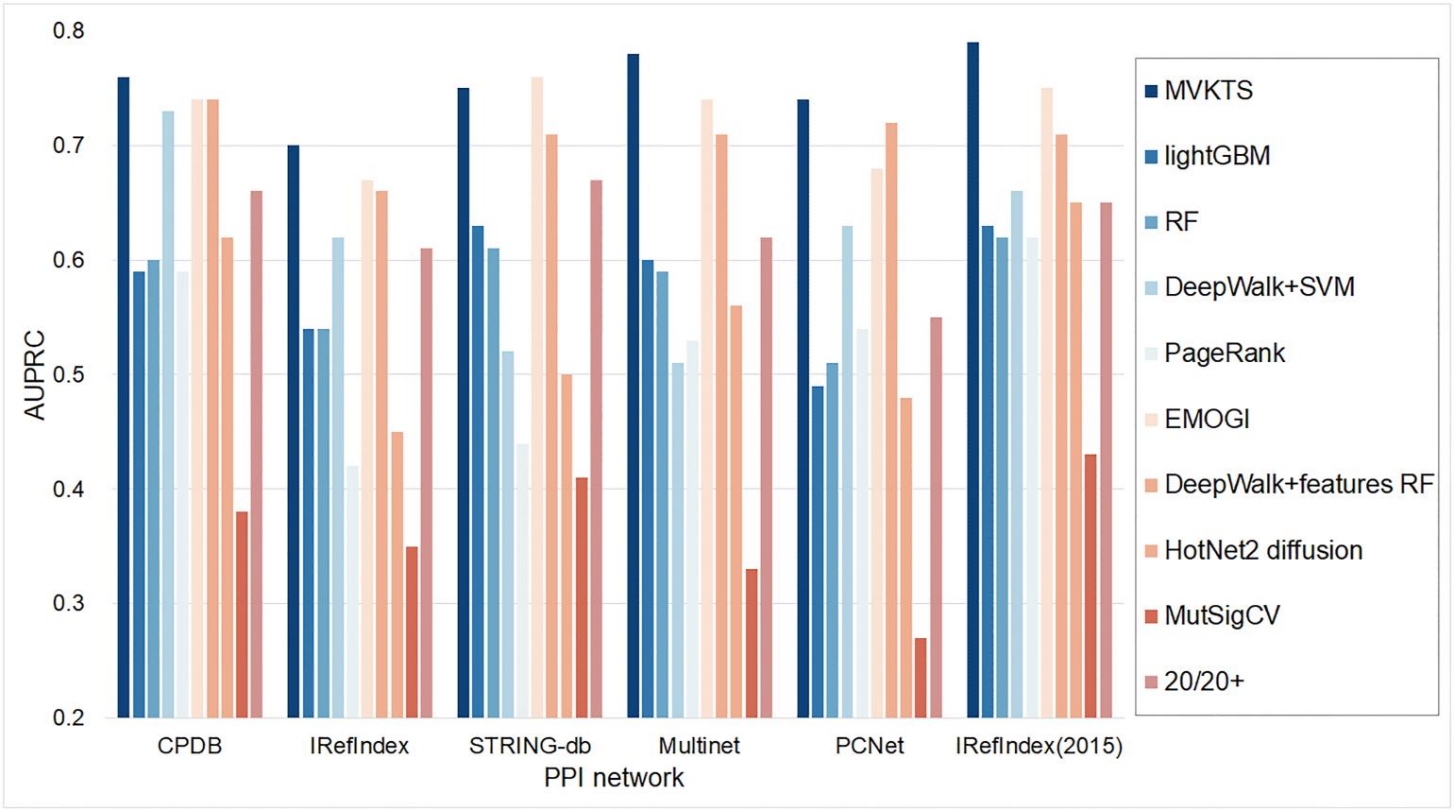
AUPRC values of MVKTS for four data type prediction methods under six PPI networks. (i) Methods based on omics features only: random forest classifier (RF) and lightGBM classifier. (ii) Pure network feature method: PageRank and DeepWalk combined with support vector machine (SVM). (iii) Methods combining omics features with network features: HotNet2 network diffusion method, DeepWalk combining RF, and EMOGI. (iv) Methods specifically designed to predict cancer genes from mutational signatures: MutSigCV and 20/20+.

To validate the effectiveness of our proposed MVKTS algorithm in recognizing cancer driver genes, we use AUPRC as a performance evaluation metric [27]. The AUPRC values are first calculated using a five‐fold cross validation process, averaging the MVKTS algorithm to evaluate performance across different PPI networks. We compare MVKTS against various cancer gene prediction methods, categorizing them into four types based on the data they utilize and calculating AUPRC for each method’s test set. The first baseline, based solely on omics features, employs both a RF and lightGBM classifier trained on all multi‐omics features for 16 cancer types [28, 29]. The second pure network feature baseline uses PageRank and DeepWalk algorithms, which relies solely on PPI networks for node prediction. To meaningfully use DeepWalk for node prediction, we input the output network features into a support vector machine (SVM) for node ranking [30]. The third type of baseline combines omics and network features, including the HotNet2 network diffusion method, a combination of DeepWalk and RF classifier method, and the EMOGI method, with HotNet2 being particularly noted for its success in identifying cancer gene modules in the last few years [31]. The fourth type of baselines are MutSigCV and 20/20+, which are widely used in cancer biology to predict cancer genes from mutational features only. Among them, the prediction method of the third baseline is closest to the MVKTS method. The driver gene scores for these methods are obtained from previous studies [19]. MVKTS outperforms nearly all other methods in the six different PPI networks (Figure 2). The performance on the STRING‐db network is better than MVKTS in EMOGI with the same feature type as MVKTS, but lower than MVKTS on all other networks. These experimental results demonstrate the effectiveness of MVKTS.

### 2.3 Ablation study of MVKTS

The MVKTS model is based on the extraction of cancer‐specific features as well as pan‐cancer features on the basis of multi‐omics features, and the cancer driver genes are predicted by integrating the extracted features. To evaluate the performance of the extracted features, we compare the prediction capabilities using the following six features (Table 1).

1. Biological features: mutation, DNA methylation, copy number variation, and gene expression data from TCGA for 16 cancers, normalized by pre‐processing to obtain multi‐omics features data.
2. Functional features: Functional feature data are obtained from multi‐omics feature data through network propagation.
3. Functional feature + distribution feature: The functional feature data obtained from multi‐omics feature data through network propagation are spliced with the distribution feature data composed of the mean‐variance kurtosis extracted from the functional feature data to further enhance the features.
4. Functional features + distribution features + SetExpan features: The functional features data obtained from the multi‐omics feature data through network propagation are spliced with the distribution features data extracted from the functional features data and SetExpan features to further enhance the features.
5. Functional feature + distribution feature + TOPSIS feature: The functional feature data obtained from multi‐omics feature data through network propagation are spliced with the distribution feature data extracted from the functional feature data and TOPSIS feature to further enhance the features.
6. Functional features + distribution features + TOPSIS features + SetExpan features: This is the MVKTS model.

**TABLE 1:** Ablation study of MVKTS.

Methods/AUPRC	CPDB	IRefIndex	STRING‐db	Multinet	PCNet	IRefIndex_(2015)_
Biological features	0.5943	0.5410	0.6296	0.5972	0.4926	0.6349
Functional features	0.7460	0.6685	0.7312	0.7547	0.7254	0.7744
Functional + Distribution features	0.7538	0.6774	0.7313	0.7643	0.727	0.7730
Functional + Distribution + SetExpan features	0.7486	0.6788	0.7352	0.7629	0.7281	0.7776
Functional + Distribution + TOPSIS features	0.7562	0.6878	0.7352	0.7588	0.7306	0.7807
MVKTS	**0.7626**	**0.6963**	**0.7515**	**0.7755**	**0.7367**	**0.7859**

It is observed that the functional features obtained by the network propagation algorithm, which merges the omics features with the network features, significantly enhances the performance of the model. While the addition of distribution features, TOPSIS features, or SetExpan features individually does not improve the model, their combination leads to notable performance improvements. Finally, the AUPRC values of the MVKTS model under the combined six PPI networks are higher than their feature combination strategies [27], which underscores the effectiveness of MVKTS in advancing cancer driver gene identification by refining feature extraction.

### 2.4 Enrichment analysis of MVKTS

**FIGURE 3:**
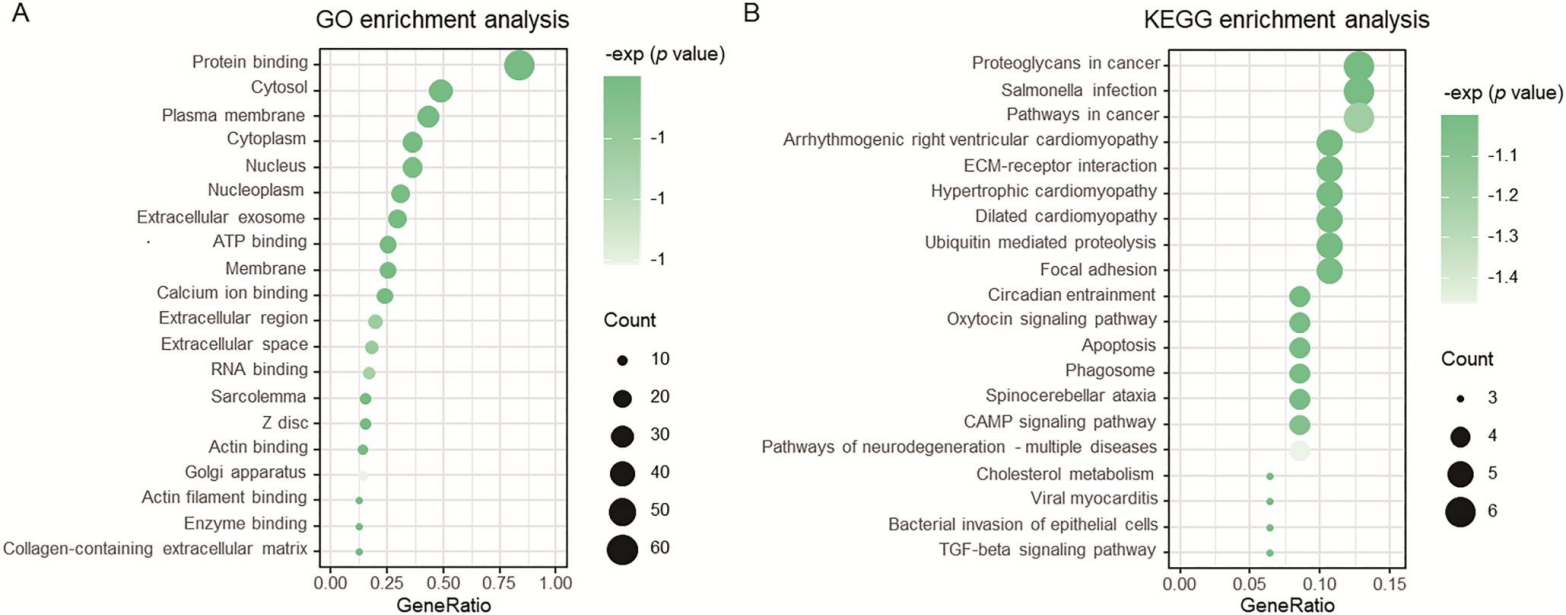
Enrichment analysis of candidate driver genes predicted by MVKTS. (A) GO terminology enrichment analysis. (B) KEGG pathway enrichment analysis.

To evaluate the biological relevance of the driver genes predicted by MVKTS, we aggregated the top 50 genes from each PPI network predicted by MVKTS. After excluding the positive sample genes used for training and testing, we identified 72 candidate driver genes. We performed GO and pathway enrichment analyses on these 72 genes using KOBAS [32]. The GO enrichment analysis (Figure 3) revealed that the 72 candidate driver genes predominantly function in “protein binding”, “cytosol”, “plasma membrane” and “cytoplasm”. Among the top 20 pathways, the most prominent pathways are “proteoglycans in cancer”, “Salmonella infection”, and “pathways in cancer”, indicating a strong association between the MVKTS predicted candidate driver genes with cancer.

## 3 DISCUSSION

In this study, we introduce MVKTS, a novel framework designed to identify pan‐cancer driver genes. MVKTS extracts functional features from the biological and PPI network features, and then further extracts useful information from the functional features to expand the gene features. The experimental results show that MVKTS consistently outperforms baseline models in identifying pan‐cancer driver genes. The ablation experimental study shows that the MVKTS models that combine multiple extraction methods achieve higher AUPRC values than those with other feature extraction combinations. The superior performance of the MVKTS model can be attributed to two main factors: firstly, the use of a network propagation algorithm to adequately structure the biological and topological gene features; secondly, the further extraction of functional features by MVKTS results in more effective gene features for pan‐cancer prediction.

Despite its effectiveness in identifying pan‐cancer driver genes, MVKTS faces limitations, such as the practical challenge that medical practitioners may not always have datasets encompassing all types of data required by the model. Additionally, while MVKTS currently concatenates features extracted by various methods, integrating advanced gene feature fusion techniques to combine four types of omics data could potentially enhance the identification of pan‐cancer driver genes [33, 34, 35]. The application of weighted fusion can also be considered to further improve the performance of MVKTS [36, 37].

## 4 MATERIALS AND METHODS

### 4.1 Datasets

We utilized the same four omics datasets and PPI network as EMOGI [19]. A brief description of these data is given. We only considered the interactions of high‐confidence protein interaction networks, including CPDB [38], STRING‐db [39], Multinet [40], older versions of IRefIndex (v.9.0) [41], the latest version of IRefIndex (v.15.0), and PCNet [42]. We used mutation, gene expression, DNA methylation, and copy number variation data from TCGA for 16 cancers, which involves collecting 29,446 samples for this study. We included in our analysis only cancer types for which DNA methylation data in tumor and normal tissue were available on TCGA, and where preprocessed batch effect‐corrected gene expression data existed [19, 43].

### 4.2 Data preprocessing

For each of the 16 cancer types, we calculated biological indicators of the genes under each cancer type [19]. The mutation feature is calculated as the mean of copy number variants and single nucleotide variants for all samples of each cancer type. The DNA methylation feature is obtained by averaging the dissimilarity of the methylation signal between normal and tumor samples for all 16 cancer samples [19]. The definition is as follows:

(1) $$dm_{i}^{c}=\frac{1}{\left|S_{c}\right|}\sum_{s\in S_{c}}\left(\beta_{si}^{t}-\beta_{si}^{n}\right)$$

where $dm_{i}^{c}$ is the DNA methylation value for each gene *i* in type *c* cancer [44]. $\beta_{si}^{t}$ and $\beta_{si}^{n}$ are the methylation signals in tumor samples and matched normal samples. *S*_*c*_ is the sample set of cancer [19, 44].

The gene expression feature is obtained by log_2_ fold difference between the expression values of 16 cancers and matched normal samples [44], which is then averaged across all samples. The copy number variation feature counts the number of times all genes are amplified or missing in a particular cohort [19]. Finally, the values from different omics data of different sizes are subjected to row min‐max normalization [19]. Each gene has four biological feature vectors consisting of a pan‐cancer mutation matrix with *N* rows and 16 columns, a pan‐cancer DNA methylation matrix with *N* rows and 16 columns, a pan‐cancer gene expression matrix with *N* rows and 16 columns, and a pan‐cancer copy number variation matrix with *N* rows and 16 columns.

The same pan‐cancer positive and negative samples as EMOGI are used in this study [19], where the positive samples include the NCG [8], a superset of the COSMIC CGC and high‐confidence genes mined from the literature. The list of negative samples is based on all samples with the deletion of genes from the NCG, COSMIC CGC, Online Mendelian Inheritance in Man database, and Kyoto Encyclopedia of Genes and Genomes cancer pathway [45, 46]. For example, in the Multinet PPI network, 790 positive samples, and 3709 negative samples were used for training. The numbers of positive and negative samples in the six PPI networks are shown in Table 2.

**TABLE 2:** Number of positive and negative samples.

PPI	Positive	Negative	Total
CPDB	796	2185	13,627
IRefIndex	836	4056	17,013
STRING‐db	783	2415	13,179
Multinet	790	3709	14,398
PCNet	859	5483	19,781
IRefIndex_(2015)_	785	1973	12,129

### 4.3 Network propagation

In order to enhance the weak functional similarity between genes in the same PPI network and obtain the functional features of genes [47], we used a network propagation algorithm with uncertainty coefficients [14]. Network propagation can associate functions between genes of any omics type frequency in the same PPI network, especially those of lower frequency genes [48].

For each cancer type, we used a network propagation algorithm to smooth the effects of the biological features of genes on other genes in the PPI network [14]. The network propagation algorithm is a random walk process. Given a cancer type *s* ∈ *S*, the equation is as follows:

(2) $$F^{t}=\alpha L^{\prime }F^{t-1}+\left(1-\alpha \right)Y$$

where *F* ^0^ = *Y* is a kind of omics information of cancer type *s*. Taking the mutation matrix as an example, *F* ^0^ = *Y* corresponds to a column in the mutation matrix. *L* is the adjacency matrix of the protein interaction network. *α* is a parameter that controls the diffusion distance of the network [49]. The network propagation process is iterative until *F* ^*t*^ converges, with the convergence condition being ${\|F^{t}-F^{t-1}\|}_{2}<10^{-6}$ [49]. The final obtained matrix *F* ^*t*^ represents the post‐propagation gene profile of cancer type *s* [49].

The network propagation algorithm is applied to all omics data to obtain four matrices, which are then connected to obtain the functional matrix *F*, each column of which corresponds to the cancer type features after propagation under the corresponding omics data.

### 4.4 Construction of gene enhancement features

After obtaining the genes’ functional features by network propagation, we extracted the genes’ enhanced features from the functional features, which include the distribution [26], TOPSIS, and SetExpan features of genes [20, 23]. It should be noted that each feature is extracted under different omics data, that is, functional features are extracted before concatenation. For each gene, enhanced features were extracted from 16 types of cancer data and combined into pan‐cancer type data to effectively ameliorate the identification of pan‐cancer driver genes.

#### 4.4.1 Distribution features of genes

The distribution features of genes are extracted from the concentration trend, dispersion degree, and distribution shape of gene functional features [26]. First, we calculated the mean value of the functional features from 16 cancer types for each gene as the concentrated trend of distribution features, which represents its general level in 16 cancer types. Since this was calculated using different omics data, 4 columns of concentrated trend features were obtained. The formula is shown as follows:

(3) $$\mu =\frac{\sum x_{ij}}{N}$$

where *x*_*ij*_ denotes the value of the *i*th gene under the *j*th cancer type and *N* denotes the number of cancer types.

The degree of data dispersion is another important feature of data distribution. We calculated the variance of the functional features of the 16 cancer types for each gene as the dispersion of the distribution features, representing its mean difference from the mean in the 16 cancer types. This was subsequently calculated with different omics data, resulting in 4 columns of dispersion degree features. The variance formula is as follows:

(4) $$\sigma^{2}=\frac{\sum_{i=1}^{N}{\left(x_{ij}-\mu \right)}^{2}}{N}$$

where *μ* denotes the mean value of genes under all cancer types.

Finally, kurtosis representation was used for the distribution shape. We then calculated the kurtosis of the functional features of the 16 cancer types in each gene under different omics data and obtained 4 columns of distribution shape features. The kurtosis of a distribution is defined as:

(5) $$k=\frac{E{\left(x_{ij}-\mu \right)}^{4}}{\sigma^{4}}$$

where *σ* is the standard deviation of genes under all cancer types and *E*(∙) represents the expected value.

#### 4.4.2 TOPSIS features of genes

Technique for order preference by similarity to ideal solution (TOPSIS) is a widely used comprehensive scoring method that fully utilizes all information from the 16 cancer types of the four omics data [20, 22], resulting in an accurate reflection of the disparity between various cancer types and a pan‐cancer gene ranking. We first calculated the optimal and inferior solutions for all pan‐cancer types [21]. Next, we evaluated the combined distance of any cancer type from the ideal and worst solution [50]. For example, if a gene is close to the ideal solution and far from the worst [50], we can assume that it has a high pan‐cancer score for that cancer type. Lastly, the close fitting progress of any cancer type to the ideal solution, which indicates its proximity to the optimal solution and distance from the least optimal solution, is calculated to rank the scores of each cancer type and select the best pan‐cancer gene ranking.

Prior to TOPSIS feature extraction, we normalized and weighted the functional feature matrix to obtain a weighted canonical matrix [51]. The entropy weighting formula is shown as follows:

(6) $$H_{j}=-\ln{\left(n\right)}^{-1}\sum_{i=1}^{n}\frac{C_{ij}}{\sum_{i=1}^{n}C_{ij}}\ln\frac{C_{ij}}{\sum_{i=1}^{n}C_{ij}}$$

(7) $$w_{j}=\frac{1-H_{j}}{m-\sum_{j=1}^{m}H_{j}}$$

where *H*_*j*_ denotes the entropy value, *w*_*j*_ denotes the entropy weight, *n* denotes the number of genes, *m* denotes the number of cancer types, and *C*_*ij*_ denotes the value of the *i*th gene under the *j*th cancer type.

TOPSIS calculates the optimal solution $I^{+}$ and the inferior solution $I^{-}$ [20]. The formula is as follows:

(8) $$\left\{\begin{array}{l} I_{j}^{+}=\max\;I_{j}\\ I_{j}^{-}=\min\;I_{j} \end{array}\right.$$

where *I*_*j*_ denotes the *j*th column of the weighted norm matrix.

TOPSIS uses the Minkowski distance to calculate the distance *D* ^+^ for each cancer type from the optimal vector and the distance *D* ^−^ for each cancer type from the worst vector [52]. The calculation formula is as follows:

(9) $$\left\{\begin{array}{l} D^{+}={\left(\sum_{j=1}^{n}{\left|I_{j}^{+}-I_{ij}\right|}^{p}\right)}^{p^{-1}}\\ D^{-}={\left(\sum_{j=1}^{n}{\left|I_{j}^{-}-I_{ij}\right|}^{p}\right)}^{p^{-1}} \end{array}\right.$$

where *I*_*ij*_ denotes the value corresponding to the *j*th cancer type of the *i*th gene in the weighted normative matrix *I*, and *p* is a variable parameter.

Finally, the relative closeness of various cancer types to the positive ideal solution is calculated [21]. The formula is as follows:

(10) $$S_{i}=\frac{D_{i}^{-}}{D_{i}^{+}+D_{i}^{-}}$$

where *i* denotes the *i*th gene.

#### 4.4.3 SetExpan features of genes

SetExpan is an integrated approach based on unsupervised ranking that combines a set of ranked scores from different cancer types to select the optimal score in an iterative manner [23]. Specifically, the gene functional feature scores for the 16 cancer types are individually scored according to its average inverse rank among all the rankings.

### 4.5 lightGBM classification model

We used the classification algorithm lightGBM, a framework for implementing the gradient boosted decision tree algorithm [53, 54], to classify genes [24]. The goal is to construct a k‐width histogram while deconstructing consecutive floating point eigenvalues into k integers. In gene classification, we used the same positive and negative samples as EMOGI [19].

In this study, we chose the area under the precision‐recall curve as an evaluation metric to evaluate classification performance [55]. We calculated the precision rate and recall rate by changing the threshold value. The formulas are as follows:

(11) $$\left\{\begin{array}{l} \;\text{Precision}=\frac{TP}{TP+FP}\\ \;\text{Recall}=\frac{TP}{TP+FN} \end{array}\right.$$

where *FN* and *FP* are the number of incorrectly identified negative and positive samples, *TN* and *TP* are the number of correctly identified negative and positive samples.

## AUTHOR CONTRIBUTIONS

**Xiaomeng Xue**: Data curation; formal analysis; methodology; software; visualization; writing – original draft preparation. **Feng Li**: Conceptualization; funding acquisition; resources; supervision; writing – review and editing. **Junliang Shang**: Funding acquisition; writing – review and editing. **Lingyun Dai**: Data curation; formal analysis; software; supervision. **Daohui Ge**: Data curation; conceptualization; resources; supervision. **Qianqian Ren**: Data curation; formal analysis; supervision.

## CONFLICT OF INTEREST STATEMENT

The authors Xiaomeng Xue, Feng Li, Junliang Shang, Lingyun Dai, Daohui Ge, and Qianqian Ren declare that they have no conflict of interest or financial conflicts to disclose.

## ETHICS STATEMENT

This article does not contain any studies with human or animal materials performed by any of the authors.

## References

[1] Bray F , Ren J‐S , Masuyer E , Ferlay J . Global estimates of cancer prevalence for 27 sites in the adult population in 2008. Int J Cancer. 2013;132(5):1133–1145. 22752881 10.1002/ijc.27711

[2] Hanahan D , Weinberg RA . Hallmarks of cancer: the next generation. Cell. 2011;144(5):646–674. 21376230 10.1016/j.cell.2011.02.013

[3] Dinstag G , Shamir R . Prodigy: personalized prioritization of driver genes. Bioinformatics. 2020;36(6):1831–1839. 31681944 10.1093/bioinformatics/btz815 PMC7703777

[4] Garraway LA , Lander ES . Lessons from the cancer genome. Cell. 2013;153(1):17–37. 23540688 10.1016/j.cell.2013.03.002

[5] Ledford H . The cancer genome challenge. Nature. 2010;464(7291):972–974. 20393534 10.1038/464972a

[6] Weinstein JN , Collisson EA , Mills GB , Shaw KRM , Ozenberger BA , Ellrott K , et al. The cancer genome atlas pan‐cancer analysis project. Nat Genet. 2013;45(10):1113–1120. 24071849 10.1038/ng.2764 PMC3919969

[7] Zhang J , Bajari R , Andric D , Gerthoffert F , Lepsa A , Nahal‐Bose H , et al. The international cancer genome consortium data portal. Nat Biotechnol. 2019;37(4):367–369. 30877282 10.1038/s41587-019-0055-9

[8] Repana D , Nulsen J , Dressler L , Bortolomeazzi M , Venkata SK , Tourna A , et al. The network of cancer genes (NCG): a comprehensive catalogue of known and candidate cancer genes from cancer sequencing screens. Genome Biol. 2019;20(1):1. 30606230 10.1186/s13059-018-1612-0 PMC6317252

[9] Sondka Z , Bamford S , Cole CG , Ward SA , Dunham I , Forbes SA . The cosmic cancer gene census: describing genetic dysfunction across all human cancers. Nat Rev Cancer. 2018;18(11):696–705. 30293088 10.1038/s41568-018-0060-1 PMC6450507

[10] Guo H , Lv X , Li Y , Li M . Attention‐based gcn integrates multi‐omics data for breast cancer subtype classification and patient‐specific gene marker identification. Brief Funct Genomics. 2023;22(5):463–474. 37114942 10.1093/bfgp/elad013

[11] Tamborero D , Gonzalez‐Perez A , Lopez‐Bigas N . Oncodriveclust: exploiting the positional clustering of somatic mutations to identify cancer genes. Bioinformatics. 2013;29(18):2238–2244. 23884480 10.1093/bioinformatics/btt395

[12] Lawrence MS , Stojanov P , Polak P , Kryukov GV , Cibulskis K , Sivachenko A , et al. Mutational heterogeneity in cancer and the search for new cancer‐associated genes. Nature. 2013;499(7457):214–218. 23770567 10.1038/nature12213 PMC3919509

[13] Tokheim CJ , Papadopoulos N , Kinzler KW , Vogelstein B , Karchin R . Evaluating the evaluation of cancer driver genes. Proc Natl Acad Sci USA. 2016;113(50):14330–14335. 27911828 10.1073/pnas.1616440113 PMC5167163

[14] Cowen L , Ideker T , Raphael BJ , Sharan R . Network propagation: a universal amplifier of genetic associations. Nat Rev Genet. 2017;18(9):551–562. 28607512 10.1038/nrg.2017.38

[15] Page L , Brin S , Motwani R , Winograd T . The pagerank citation ranking: bringing order to the web; 1998; ID: 1508503.

[16] Leiserson MDM , Vandin F , Wu H‐T , Dobson JR , Eldridge JV , Thomas JL , et al. Pan‐cancer network analysis identifies combinations of rare somatic mutations across pathways and protein complexes. Nat Genet. 2015;47(2):106–114. 25501392 10.1038/ng.3168 PMC4444046

[17] Perozzi B , Al‐Rfou R , Skiena S . Deepwalk: online learning of social representations. In: Proceedings of the 20th ACM SIGKDD international conference on knowledge discovery and data mining; 2014. p. 701–710.

[18] Zhang S‐W , Xu J‐Y , Zhang T . Dgmp: identifying cancer driver genes by jointing DGCN and MLP from multi‐omics genomic data. Dev Reprod Biol. 2022;20(5):928–938. 10.1016/j.gpb.2022.11.004 PMC10025764 36464123

[19] Schulte‐Sasse R , Budach S , Hnisz D , Marsico A . Integration of multiomics data with graph convolutional networks to identify new cancer genes and their associated molecular mechanisms. Nat Mach Intell. 2021;3(6):513–526.

[20] Pavić Z , Novoselac V . Notes on topsis method. Int J Res Eng Sci. 2013.

[21] Chen P . Effects of the entropy weight on topsis. Expert Syst Appl. 2021;168:114186.

[22] Shih H‐S , Shyur H‐J , Lee ES . An extension of topsis for group decision making. Math Comput Model. 2007;45(7‐8):801–813.

[23] Shen J , Wu Z , Lei D , Shang J , Ren X , Han J . Setexpan: corpus‐based set expansion via context feature selection and rank ensemble. In: Machine learning and knowledge discovery in databases. Springer International Publishing; 2017. p. 288–304.

[24] Chen X , Liu X . A weighted bagging lightgbm model for potential lncrna‐disease association identification. In: Bio‐inspired computing: theories and applications. Springer Singapore; 2018. p. 307–314.

[25] Collier O , Stoven V , Vert J‐P . Lotus: a single‐ and multi‐task machine learning algorithm for the prediction of cancer driver genes. PLoS Comput Biol. 2019;15(9):e1007381. 31568528 10.1371/journal.pcbi.1007381 PMC6786659

[26] Gumpinger AC , Lage K , Horn H , Borgwardt K . Prediction of cancer driver genes through network‐based moment propagation of mutation scores. Bioinformatics. 2020;36(Suppl_1):508–515. 10.1093/bioinformatics/btaa452 PMC7355253 32657361

[27] Boyd K , Eng KH , Page CD . Area under the precision‐recall curve: point estimates and confidence intervals. In: Machine learning and knowledge discovery in databases. Springer Berlin Heidelberg; 2013. p. 451–466.

[28] Ziegler A , Koenig IR . Mining data with random forests: current options for real‐world applications. Wiley Interdiscip Rev Data Min Knowl Discov. 2014;4(1):55–63.

[29] Bao W , Cui Q , Chen B , Yang B . Phage_unir_lgbm: phage virion proteins classification with unirep features and lightgbm model. Comput Math Methods Med. 2022;2022:1–8. 10.1155/2022/9470683 PMC9033350 35465015

[30] Huang S , Cai N , Pacheco PP , Narandes S , Wang Y , Xu W . Applications of support vector machine (SVM) learning in cancer genomics. Cancer Genomics Proteomics. 2018;15:41–51. 29275361 10.21873/cgp.20063 PMC5822181

[31] Kristensen VN , Lingjoerde OC , Russnes HG , Vollan HKM , Frigessi A , Borresen‐Dale A‐L . Principles and methods of integrative genomic analyses in cancer. Nat Rev Cancer. 2014;14(5):299–313. 24759209 10.1038/nrc3721

[32] Xie C , Mao X , Huang J , Ding Y , Wu J , Dong S , et al. Kobas 2.0: a web server for annotation and identification of enriched pathways and diseases. Nucleic Acids Res. 2011;39(Suppl l_2):W316–W322. 21715386 10.1093/nar/gkr483 PMC3125809

[33] Ma T , Zhang A . Affinity network fusion and semi‐supervised learning for cancer patient clustering. Methods. 2018;145:16–24. 29807109 10.1016/j.ymeth.2018.05.020

[34] Zhao W , Gu X , Chen S , Wu J , Zhou Z . Modig: integrating multi‐omics and multi‐dimensional gene network for cancer driver gene identification based on graph attention network model. Bioinformatics. 2022;38(21):4901–4907. 36094338 10.1093/bioinformatics/btac622

[35] Shi X , Teng H , Shi L , Bi W , Wei W , Mao F , et al. Comprehensive evaluation of computational methods for predicting cancer driver genes. Briefings Bioinf. 2022;23(2):bbab548. 10.1093/bib/bbab548 PMC8921613 35037014

[36] Ren TY , Ye FF , Yang LH , Liu J , Wang Y . Dynamic rule activation method based on activation factor for extended belief rule‐based systems. In: 2021 16th international conference on intelligent systems and knowledge engineering (ISKE); 2021. p. 82–86.

[37] Wu H , Chen Z , Wu Y , Zhang H , Liu Q . Integrating protein‐protein interaction networks and somatic mutation data to detect driver modules in pan‐cancer. Interdiscipl Sci Comput Life Sci. 2022;14(1):151–167. 10.1007/s12539-021-00475-y 34491536

[38] Kamburov A , Pentchev K , Galicka H , Wierling C , Lehrach H , Herwig R . Consensuspathdb: toward a more complete picture of cell biology. Nucleic Acids Res. 2011;39(Suppl l_1):D712–D717. 21071422 10.1093/nar/gkq1156 PMC3013724

[39] Szklarczyk D , Gable AL , Lyon D , Junge A , Wyder S , Huerta‐Cepas J , et al. String v11: protein‐protein association networks with increased coverage, supporting functional discovery in genome‐wide experimental datasets. Nucleic Acids Res. 2019;47(D1):D607–D613. 30476243 10.1093/nar/gky1131 PMC6323986

[40] Khurana E , Fu Y , Chen J , Gerstein M . Interpretation of genomic variants using a unified biological network approach. PLoS Comput Biol. 2013;9(3):e1002886. 23505346 10.1371/journal.pcbi.1002886 PMC3591262

[41] Razick S , Magklaras G , Donaldson IM . Irefindex: a consolidated protein interaction database with provenance. BMC Bioinf. 2008;9(1):405. 10.1186/1471-2105-9-405 PMC2573892 18823568

[42] Huang JK , Carlin DE , Yu MK , Zhang W , Kreisberg JF , Tamayo P , et al. Systematic evaluation of molecular networks for discovery of disease genes. Cell Systems. 2018;6(4):484–495. 29605183 10.1016/j.cels.2018.03.001 PMC5920724

[43] Wang Q , Armenia J , Zhang C , Penson AV , Reznik E , Zhang L , et al. Unifying cancer and normal rna sequencing data from different sources. Sci Data. 2018;5(1):180061. 29664468 10.1038/sdata.2018.61 PMC5903355

[44] Peng W , Wu R , Dai W , Ning Y , Fu X , Liu L , et al. Mirna‐gene network embedding for predicting cancer driver genes. Brief Funct Genomics. 2023;22(4):341–350. 36752023 10.1093/bfgp/elac059

[45] McKusick VA . Mendelian inheritance in man and its online version, omim. Am J Hum Genet. 2007;80(4):588–604. 17357067 10.1086/514346 PMC1852721

[46] Ogata H , Goto S , Sato K , Fujibuchi W , Bono H , Kanehisa M . Kegg: Kyoto encyclopedia of genes and genomes. Nucleic Acids Res. 1999;27(1):29–34. 9847135 10.1093/nar/27.1.29 PMC148090

[47] Xiang J , Zhang N‐R , Zhang J‐S , Lv X‐Y , Li M . Prgefne: predicting disease‐related genes by fast network embedding. Methods. 2021;192:3–12. 32610158 10.1016/j.ymeth.2020.06.015

[48] Vanunu O , Magger O , Ruppin E , Shlomi T , Sharan R . Associating genes and protein complexes with disease via network propagation. PLoS Comput Biol. 2010;6(1):e1000641. 20090828 10.1371/journal.pcbi.1000641 PMC2797085

[49] Li F , Gao L , Wang B . Detection of driver modules with rarely mutated genes in cancers. IEEE ACM Trans Comput Biol Bioinf. 2020;17(2):390–401. 10.1109/TCBB.2018.2846262 29994261

[50] Zhang L‐c , Li C‐j , Yu Z‐l . Dynamic web service selection group decision‐making based on heterogeneous QOS models. J China Univ Posts Telecommun. 2012;19(3):80–90.

[51] Li Z , Luo Z , Wang Y , Fan G , Zhang J . Suitability evaluation system for the shallow geothermal energy implementation in region by entropy weight method and topsis method. Renew Energy. 2022;184:564–576.

[52] Xu H , Zeng W , Zeng X , Yen GG . An evolutionary algorithm based on minkowski distance for many‐objective optimization. IEEE Trans Cybern. 2019;49(11):3968–3979. 30059330 10.1109/TCYB.2018.2856208

[53] Chen T , Guestrin C . Xgboost: a scalable tree boosting system. In: Proceedings of the 22nd ACM SIGKDD international conference on knowledge discovery and data mining; 2016. p. 785–794.

[54] Rao H , Shi X , Rodrigue AK , Feng J , Xia Y , Elhoseny M , et al. Feature selection based on artificial bee colony and gradient boosting decision tree. Appl Soft Comput. 2019;74:634–642.

[55] Borji A , Cheng M‐M , Jiang H , Li J . Salient object detection: a benchmark. IEEE Trans Image Process. 2015;24(12):5706–5722. 26452281 10.1109/TIP.2015.2487833

